# Editoral: Antibacterial, anti-inflammatory, and ROS-responsive drug delivery nanoplatforms

**DOI:** 10.3389/fchem.2022.1124486

**Published:** 2023-01-04

**Authors:** Zhenhua Chen, Botao Song, Xuelian Huang, Lin Sang

**Affiliations:** ^1^ Jinzhou Medical University, Jinzhou, Liaoning, China; ^2^ Northwest University, Xi’an, Shaanxi, China; ^3^ University of Washington, Seattle, WA, United States; ^4^ Dalian University of Technology, Dalian, China

**Keywords:** antibacterial, anti-inflammatory, oxidative stress, drug delivery, nanoplatform

Bacterial infection, excessive inflammation, and excessive oxidative stress are widespread in the microenvironments of many infectious lesions and wounds. These problems commonly occur during the repair of arthritis cartilage, gout, diabetic wounds, and chronically infected wounds. To solve excessive inflammation, excessive oxidative stress, and related long-term chronic pain, hormonal drugs and non-steroidal drugs are widely used in clinical practice; however, the damage to the gastrointestinal tract, liver, and kidney function caused by these drugs cannot be ignored. Regarding these Research Topic, antibacterial, anti-inflammatory, and reactive oxygen species (ROS)-responsive drug delivery nanoplatforms are very attractive and promising.

Yin et al. reported berberine-modified ZnO nanocolloids loaded with a hydrogel (ZnO-Ber/H) for use as a nanoplatform in diabetic wound healing. ZnO-Ber/H significantly downregulated the levels of TNF-α, IL-1β, and IL-6 inflammatory factors by 72.8%, 55%, and 71%, respectively. Meanwhile, ZnO-Ber/H upregulated the anti-oxidative stress factors Nrf2, HO-1, and NQO1 by 4.65-fold, 2.49-fold, and 2.56-fold, respectively, facilitating the construction of a microenvironment for tissue repair. In addition, ZnO-Ber/H enhanced the expression of the vascular-associated factors VEGF and CD31 by 3.9-fold and 3.2-fold, respectively, which in turn promoted tissue granulation and neovascularization. *In vivo* treatment showed that the use of ZnO-Ber/H resulted in a 92.9% wound healing rate in a 15-day treatment cycle, significantly shortening the diabetic wound healing cycle.

Lv et al. designed a coordination compound coated with hyaluronic acid (HA), based on apigenin (API) and Mn^2+^, for the treatment of ulcerative colitis caused by sodium dextran sulfate. API-Mn(II)@HA effectively repaired the intestinal barrier by modulating typical and atypical inflammatory signaling pathways, significantly improving damaged colon tissue by regulating inflammatory factor expression. HA encapsulation improved the targeting effect of API-Mn(II)@HA on colonic inflammation, effectively reducing side effects and improving the bioavailability. The results reveal that API-Mn(II)@HA exhibited stronger anti-inflammatory effects than the reference drug 5-ASA and effectively alleviated ulcerative colitis with a good biosafety profile.

Ma et al. proposed a gentamicin-loaded type I collagen/hyaluronic acid multilayer modified titanium coating [TC-AA(C/H)_6_-G] for reducing the risk of infection after bone implant placement. TC-AA(C/H)_6_-G achieved an efficient loading of gentamicin (537.22 ± 29.66 g) and good controlled release *in vivo* (240 h retardation time) through encapsulation of type I collagen and hyaluronic acid multilayers. The results show that the TC-AA(C/H)_6_-G coating significantly inhibited colonization and biofilm formation and prevented medullary infections caused by *S. aureus*. The slow-release properties of the TC-AA(C/H)_6_-G coating effectively address the shortcomings of high local drug concentrations and the low utilization of conventional drug delivery methods, making it a potentially attractive option for preventing implant infections.

Gao et al. reported epigallocatechin gallate (EGCG)-loaded gold nanocages (AuNCs) as anticancer nanoplatforms. AuNCs-EGCG exhibited near-infrared (NIR)–responsive properties, a maximum photothermal temperature of 41.4°C, and good photothermal stability. Under NIR irradiation, AuNCs-EGCG achieved synergistic inhibition of hepatocellular carcinoma cell proliferation *via* the photothermal effect and the release of EGCG. NIR-responsive AuNCs-EGCG significantly upregulated caspase-3 almost 2-fold and downregulated Bcl-2 almost .33-fold, which effectively inhibited HepG2 cell proliferation through the mitochondrial pathway.

The goal of this Research Topic is to explore and obtain multifunctional nanoplatform materials with antibacterial, anti-inflammatory, and oxidative stress-regulating functions, which can be applied for diabetic wound repair, arthritis cartilage repair, and other relevant circumstances. This Research Topic aims to highlight the most advanced achievements in the medicinal and pharmaceutical chemistry of nanoplatform materials, which should inspire and guide the future direction of this field.

